# School Programs and Characteristics and Their Influence on Student BMI: Findings from Healthy Passages

**DOI:** 10.1371/journal.pone.0083254

**Published:** 2014-01-13

**Authors:** Tracy K. Richmond, Marc N. Elliott, Luisa Franzini, Ichiro Kawachi, Margaret O. Caughy, M. Janice Gilliland, Courtney E. Walls, Frank A. Franklin, Richard Lowry, Stephen W. Banspach, Mark A. Schuster

**Affiliations:** 1 Division of Adolescent Medicine, Boston Children's Hospital, Boston, Massachusetts, United States of America; 2 Statistics, RAND Corporation, Santa Monica, California, United States of America; 3 Division of Management, Policy, and Community Health, The University of Texas School of Public Health/Blue Cross and Blue Shield of Texas Research Program in Payment Systems and Policies, Houston, Texas, United States of America; 4 Department of Social and Behavioral Sciences, Harvard School of Public Health, Boston, Massachusetts, United States of America; 5 University of Texas School of Public Health, Dallas, Texas, United States of America; 6 Birmingham, Alabama, United States of America; 7 Epidemiology, Decision Resources, Burlington, Massachusetts, United States of America; 8 Department of Maternal and Child Health, School of Public Health, University of Alabama at Birmingham, Birmingham, Alabama, United States of America; 9 Centers for Disease Control and Prevention, Atlanta, Georgia, United States of America; 10 Department of Medicine, Boston Children's Hospital, Boston, Massachusetts, United States of America; Children's National Medical Center, Washington, United States of America

## Abstract

**Background:**

Little is known about the contribution of school contextual factors to individual student body mass index (BMI). We set out to determine if school characteristics/resources: (1) are associated with student BMI; (2) explain racial/ethnic disparities in student BMI; and (3) explain school-level differences in student BMI.

**Methods:**

Using gender-stratified multi-level modeling strategies we examined the association of school characteristics/resources and individual BMI in 4,387 5^th^ graders in the Healthy Passages Longitudinal Study of Adolescent Health. Additionally, we examined the association of race/ethnicity and individual BMI as well as the between-school variance in BMI before and after adding individual and school characteristics to test for attenuation.

**Results:**

The school-level median household income, but not physical activity or nutrition resources, was inversely associated with female BMI (β = −0.12, CI: −0.21,−0.02). Neither school demographics nor physical activity/nutrition resources were predictive of individual BMI in males. In Black females, school characteristics attenuated the association of race/ethnicity and BMI. Individual student characteristics—not school characteristics/resources-reduced the between-school variation in BMI in males by nearly one-third and eliminated it in females.

**Conclusions:**

In this cohort of 5^th^ graders, school SES was inversely associated with female BMI while school characteristics and resources largely explained Black/White disparities in female weight status. Between-school differences in average student weight status were largely explained by the composition of the student body not by school characteristics or programming.

## Background

Numerous interventions aimed at reversing the childhood obesity epidemic have focused on schools. Schools are a logical place to intervene because children and adolescents spend at least one third of their annual waking hours in school, may consume as much as 50% of their daily calories at school, and are potentially exposed to numerous opportunities for physical activity through school [1]–[3]. However, schools do not currently provide students equal opportunities for healthy choices. Schools have rapidly re-segregated since mandatory racial/ethnic desegregation laws were repealed in the early 1990s [4], [5]. This re-segregation likely has health implications as studies have demonstrated poorer nutritional and physical activity offerings in schools with high concentrations of racial/ethnic minority and low-income students [1], [3].

Several studies have demonstrated differences between high schools in average student weight status; in at least two of these studies, between-school differences were not fully explained by the divergent demographics of the student populations [6], [7]. Questions remain, however, whether school-level differences in weight status exist in elementary schools and if physical activity and nutrition programming or demographics can explain these differences in weight status. Additionally, it is unclear whether school characteristics can partially explain racial/ethnic differences in weight status.

Our objectives were to determine if: 1) school characteristics or resources are associated with individual student BMI; 2) school characteristics and resources partially explain racial/ethnic disparities in individual student weight status; and 3) individual student and/or school characteristics can explain between-school differences in student BMI. We hypothesized that students attending a school with higher physical activity and nutrition resources and higher median income would have on average lower BMIs than those students attending schools with lower physical activity and nutrition resources and lower median income and that differential resources could partially explain racial/ethnic and between-school differences in student BMI.

## Methods

We used data from the Healthy Passages Longitudinal Study of Adolescent Health, a prospective cohort study of 5^th^ grade students in Birmingham, AL, Los Angeles, CA, and Houston, TX [8], [9]. Healthy Passages has a multilevel approach, collecting data from the individual andhis/her parent or caregiver, school, and neighborhood in an effort to understand the complex influences on risk and protective factors, health behaviors, and health outcomes. We used data from the baseline data collection (2004–2006).

### Participants

The sample was derived from the study population that included all fifth-grade students enrolled in public schools with an enrollment of ≥25 fifth graders in each of three geographic areas (25 contiguous public school districts in Los Angeles County, CA; 20 contiguous public school districts in and around Birmingham, AL; and the largest public school district in Houston, TX). Healthy Passages used a two-stage probability sampling strategy, described in detail elsewhere [9], to ensure adequate sample sizes of non-Hispanic Blacks, Hispanics, and non-Hispanic Whites. The 5,147 participants were nested in 112 schools within the three survey sites.

Parents provided written consent for their own as well as their child's participation; the child participant provided written assent. The Institutional Review Boards of the sites (RAND/University of California Los Angeles, University of Alabama Birmingham, University of Texas Houston branch) approved the consent process. Computer-assisted Spanish or English interviews were conducted in the participants' homes, at a study center, or another preferred location. Our research team received only de-identified data. Our analyses were approved through a waiver of the Boston Children's Hospital Institutional Review Board.

### Outcome variable

#### Body Mass Index (kg/m^2^)

Respondents' BMIs were calculated from measured height and weight. Height and weight measurements were performed by trained field interviewers. Height was measured using a portable stadiometer and measurements were recorded to 0.1 cm. Weight was measured using a portable Tanita electronic scale and was recorded to 0.1 kg. Height and weight were measured two times and averaged; if the two differed by more than a pre-specified amount (0.5 cm for height, 0.2 kg for weight), three measurements were performed and the average of the 2 measurements with the smallest difference was taken.

### Individual-level predictor variables

#### Race/ethnicity

Our primary predictor variable was student race/ethnicity. Participants and participants' parents were asked whether the student was of Hispanic/Latino ethnicity and of which racial group they were a member. When available, the participant-response to Hispanic/Latino ethnicity and participant-chosen racial group was used. Where participant-response of either ethnicity or racial group was unavailable, the parental report was used. We used U.S. Census definitions to initially classify the sample into the following mutually exclusive categories: Asian/Pacific Islander; Black; Hispanic; Multi-racial; Native American/Alaskan Native; and White. We classified any individual who checked Hispanic as Hispanic regardless of additional racial/ethnic categories indicated. We categorized anyone not choosing Hispanic but choosing more than one racial category as Multi-racial. Because of small sample sizes in groups other than Blacks, Whites, and Hispanics, we collapsed groups that consisted of Asian/Pacific Islander, Multi-racial, and Native American/Alaskan into a racial category named “Other.”

#### Socio-economic status measures

Healthy Passages attempted to interview a parent/caregiver for all participants and selected the parent/caregiver with the most knowledge about the child's health and education, usually the mother or female guardian. The parent or caregiver was asked to report all household income by choosing one of the following response categories: <$25,000; $25,000–$49,999; $50,000–$99,999; and >$100,000. They were also asked to report the highest educational level achieved by a household member, including that of step- or foster parents, siblings, grandparents, or significant others of caregivers with the following response options: less than high school graduation; high school graduate or GED; some college or 2 year degree; college graduate or beyond.

#### Parental/caregiver weight status

Objective measurements of the height and weight of the parent/caregiver participating in the interview—typically the mother or female guardian–were taken at the time of the interview. We calculated BMI from height and weight (weight (kg)/height^2^(m^2^)) and then categorized weight status as low/normal weight v. overweight/obese (BMI≥25 kg/m^2^). Where objective heights and/or weights were missing (9.5% of cases), we inserted the mean parental/caregiver BMI of the sample and added an indicator flag to indicate missing data. We constructed models with and without the mean parental/caregiver BMI substituted to assess bias and found no material difference in the findings. Thus, we present only the models with the mean parental BMI substituted for those with missing data to avoid potential bias and inefficiency from deleting participants.

#### Additional demographics

Age and gender were self-reported by participants.

### School-level predictor variables

Information regarding school policies and resources was provided by school staff members (typically the school principals or assistant principals—the school nurse or other administrative personnel completed the survey when the principal or assistant principal was unavailable).

#### Physical activity/nutrition resources

We examined a number of variables that described school physical activity and dietary resources (Table 1) individually and as part of a school physical activity/nutrition summary score. The school physical activity/nutrition summary score included the following variables: 1) Physical Education (PE) taught in school 4 or 5 days/week; 2) PE taught in school ≥36 weeks/year; 3) 5^th^ graders have regular recess; 4) physical activity taught in health class; 5) school does not have a pouring contract with a beverage company; 6) nutrition taught in health class; 7) brand name fast food items are not available in the cafeteria. In addition, we explored three additional variables school offers breakfast; an outside food company manages food service; and school participates in the USDA lunch program. We did not include these variables, however, in the summary score as we had no a priori hypothesis regarding the direction of their influence on BMI. Approximately 10% of school personnel surveyed did not answer the question regarding whether or not school nutrition was taught in school. When asked how many weeks/year PE was taught, a number of school personnel survey respondents answered more than 52 weeks, an implausible value. Due to a large degree of missing or implausible data for these two variables (total ∼10%) and because neither was associated with the outcome, we constructed the school summary score with and without these variables and found no material differences in the findings. In order to maximize the sample size and avoid bias, this study presents results with the school summary score not including weeks/year of PE and whether nutrition is taught in school. The school physical activity/nutrition summary score represents a count of the positive factors that apply to each school (range: 0–5).

**Table 1:** Bivariate associations between individual and school characteristics and individual race/ethnicity.

*Individual Variable*	White	Black	Hispanic	Other	p-value
	N = 1256	N = 1755	N = 1813	N = 321	
***Age*** a	11.2 (0.03)	11.2(0.02)	11.1(0.03)	11.0(0.03)	0.001
***Household income***					<0.001
<$25,000	9%	54%	53%	23%	
$25,000–$49,999	16%	27%	32%	27%	
$50,000–$99,999	35%	14%	11%	25%	
≥$100,000	40%	5%	4%	25%	
***Highest education level in household***					<0.001
Some HS	3%	10%	45%	2%	
HS/GED	9%	31%	24%	10%	
Some College	17%	37%	21%	29%	
College Plus	71%	23%	10%	60%	
**School-level variables**					
***Avg proportion of student body that is White***	80%	10%	10%	40%	<0.001
***Median school household income*** a	$80,427(5542)	$27,681^b^(1876)	$29,806^b^(2218)	$54,540^b^ (4406)	<0.001
***Physical Education is taught 4 or 5 days/week***	75%	56%	30%	49%	<0.001
***Physical Education is taught ≥36 weeks/year***	94%	89%	69%	86%	<0.001
***5^th^ graders have regular recess***	59%	56%	91%	90%	<0.001
***Physical activity and fitness taught in health class***	98%	99%	99%	99%	0.22
***School participates in USDA school lunch program***	69%	93%	93%	93%	0.003
***School does not have pouring contract with beverage company***	76%	47%	74%	74%	0.002
***Nutrition taught in health class***	89%	83%	89%	87%	0.54
***School serves breakfast***	73%	99%	99%	89%	<0.001
***Outside food service co. operates food service program***	15%	29%	35%	25%	0.01
***Brand name fast food items not available***	93%	93%	89%	85%	0.42
***School physical activity/nutrition resource summary score*** a	4.01 (0.16)	3.16^b^ (0.11)	3.87(0.13)	3.92(0.18)	0.049

Avg means Average.

^a^ *Values presented as mean (SE).*

b *indicates mean or percent is significantly different from that of the White group. p-values derived from overall F-test.*

As an additional sensitivity analysis, we conducted a factor analysis of the variables in the physical activity/nutrition summary score. This analysis resulted in three factors, one related to gym and recess, a second related to foods and beverages available in the cafeteria, and a final one related to the number of weeks gym was taught. None of the factors was associated with individual BMI so we present only findings related to the summary score.

#### School demographics

We controlled for additional school-level demographics, including the school administrator report of the percentage of students who were White and the school median household income aggregated from individual parent/caregiver report of individual household income as described above.

#### Site

Finally, we controlled for the survey site: Birmingham, Los Angeles, and Houston.

### Procedures/Data Analysis

We began by examining univariate distributions of the variables of interest. We then examined bivariate associations of our variables of interest with individual race/ethnicity. Because students were nested within schools in the sample we employed multi-level modeling strategies next in order to estimate both random and fixed effects at both the student and school level and to account for the effects of clustering in the data. Using STATA SE 12 [10] and employing weights to account for probabilities of selection and non-response, we constructed a series of three two-level “mixed” (random and fixed effect) linear regression models predicting individual BMI. Early on in our model building strategy we tested for the potential effect modification by gender on associations between race/ethnicity and BMI and found justification for gender-stratified modeling strategies.

We began with a null model with only school-level random effects to assess variation in BMI by school in terms of school-level variance components. In our second model we added student-level fixed effects (e.g., individual race/ethnicity) to allow us to examine the associations of individual characteristics with BMI. In our third model we added specific school-level variables (e.g., school demographics and school physical activity/nutrition resources) to the second model as well as study site fixed effects to examine the associations of school programs and characteristics with BMI (addressing Objective 1). We assessed changes in the parameter estimates for individual race/ethnicity from Model 2 to 3 with the addition of school characteristics (to address Objective 2). In order to better understand the contribution of individual and school-level characteristics to differences in school-level BMI (addressing Objective 3) we compared the change in between-school variance in BMI across Models 1, 2 and 3.

## Results

### Characteristics of schools attended by participants of different races/ethnicities

Table 1 shows the results of the bivariate relationship of the variables of interest and race/ethnicity. Schools attended by Blacks, Whites, and Hispanics differed on numerous school characteristics, including demographics and nutritional and physical activity programs.

### Multivariate analysis of individual- and school-level variables and student BMI

Tables 2 and 3 report findings from the multilevel models 1–3, stratified by gender.

**Table 2:** The association of individual and school factors with female BMI in the Healthy Passages cohort.

	Model 1 (ß coefficient)	Model 2 (ß coefficient)	Model 3 (ß coefficient)
**Fixed Effects**			
Intercept	20.62(19.96,21.29)	6.01 (−0.86, 12.88)	10.79(4.59, 17.06)
**Individual level variables**			
***Race/ethnicity***			
Hispanic		0.41 (−0.34, 1.16)	−0.04 (−0.76, 0.68)
Black		1.40(0.75, 2.06)	0.77(0.03, 1.51)
Other		−0.26(−1.16, 0.646)	−0.38 (−1.30, 0.54)
White [Ref]		0	0
***Age***		1.06(0.45, 1.67)	0.76 (0.22, 1.31)
***Household income***			
<$25,000		1.05(0.22, 1.89)	0.68 (−0.17, 1.52)
$25,000–$49,999		1.28 (0.49, 2.07)	0.72 (−0.06, 1.49)
$50,000–$99,999		0.95(0.30, 1.59)	0.73 (0.08, 1.39)
≥$100,000 [Ref]		0	0
***Highest education level in household*** a		−0.07 (−0.34, 0.20)	−0.04 (−0.30, 0.23)
***Parent is overweight or obese***		2.37(1.94, 2.81)	2.43 (1.98, 2.87)
***Parent missing weight status flag***		1.24(0.28, 2.20)	1.22(0.20, 2.25)
**School-level variables**			
***Proportion of student body that is White***			−0.60(−2.01, 0.81)
***School income (tens of thousands of dollars)***			−0.12(−0.21, −0.02)
***School Nutrition/Physical Activity Index*** b			−0.10(−0.38, 0.18)
**Site**			
Houston	0.77(−0.10, 1.63)	0.18(−0.34, 0.71)	0.35(−0.31, 1.00)
Birmingham	0.54(−0.38, 1.47)	0.23(−0.31, 0.77)	0.57(−0.01, 1.12)
Los Angeles [Ref]	0	0	0
**Random Effects**			
sigma_u	1.34(1.01, 1.65)	0	0.27(0.01, 7.40)
sigma_e e	4.93(4.66, 5.21)	4.82(4.56, 5.08)	4.67(4.42, 4.93)

^`^ Ref = Reference group; sigma_u is the standard deviation of the school-level random effect; sigma_e is the standard deviation of the individual-level random effect.

a High school levels categorized into some high school, high school graduate or received GED, some college, and college graduate or beyond.

b School nutrition/physical activity index is a count of the positive nutrition and physical activity resources/programs reported in the school.

**Table 3:** The association of individual and school factors with male BMI in the Healthy Passages cohort.

	Model 1 (ß coefficient)	Model 2 (ß coefficient)	Model 3 (ß coefficient)
**Fixed Effects**			
Intercept	21.38(20.82, 21.93)	9.86(5.07, 14.64)	9.30(3.89, 14.71)
**Individual level variables**			
***Race/ethnicity***			
Hispanic		1.47(0.77, 2.17)	1.58(0.81, 2.35)
Black		0.31(−0.36, 0.98)	0.44(−0.40, 1.27)
Other		1.30(0.23, 2.36)	1.36(0.26, 2.46)
White [Ref]		0	0
***Age***		0.79(0.36, 1.21)	0.78(0.33, 1.22)
***Household income***			
<$25,000		0.78(−0.06, 1.63)	0.82(−0.04, 1.67)
$25,000–$49,999		0.72(−0.14, 1.58)	0.79(−0.10, 1.68)
$50,000–$99,999		1.01(0.35, 1.68)	0.97(0.32, 1.63)
≥$100,000 [Ref]		0	0
***Highest education level in household*** a		−0.24(−0.51, 0.03)	−0.25(−0.54, 0.04)
***Parent is overweight or obese***		2.41(1.92, 2.90)	2.45(1.94, 2.96)
***Parent missing weight status flag***		0.35(−0.45, 1.14)	0.55(−0.29, 1.68)
**School-level variables**			
***Proportion of student body that is White***			0.54(−1.03, 2.11)
***School income (tens of thousands of dollars)***			−0.03(−0.16, 0.10)
***School Nutrition/Physical Activity Index*** b			0.13 (−0.26, 0.53)
**Site**			
Houston	0.14(−0.64, 0.92)	−0.25(−1.02, 0.52)	0.05(−0.85, 0.96)
Birmingham	−1.33(−2.07, −0.58)	−0.32(−1.18, 0.55)	−0.44(−1.41, 0.53)
Los Angeles [Ref]	0	0	0
**Random Effects**			
sigma_u	0.97(0.71, 1.31)	0.68(0.41, 1.14)	0.77(0.49, 1.20)
sigma_e	4.99(4.73, 5.26)	4.84(4.59, 5.11)	4.83(4.56, 5.11)

^`^ Ref = Reference group; sigma_u is the standard deviation of the school-level random effect; sigma_e is the standard deviation of the individual-level random effect.

a High school levels categorized into some high school, high school graduate or received GED, some college, and college graduate or beyond.

b School nutrition/physical activity index is a count of the positive nutrition and physical activity resources/programs reported in the school.

#### Association of school characteristics with individual student BMI (addressing Objective 1)

Among females, only the school level household income was inversely associated with individual student BMI (β = −0.12, CI: −0.21,−0.02). For males and females, neither the percent of the student body reported to be White nor the summary score representing nutrition/physical activity programming was associated with BMI. In males and females, although each of the ten variables describing the school physical activity and nutrition resources was tested individually in addition to testing the summary score itself, no statistically significant associations were found (data not presented for individual components of summary score).

#### Change in parameter estimates for race/ethnicity with the addition of school characteristics (addressing Objective 2)

The parameter estimates for Hispanic and Black race/ethnicity in Model 2 (without school characteristics) and Model 3 (with school characteristics added) were compared. Among females, the addition of school characteristics reduced the effect size of Black race by nearly half (Model 2: β = 1.40, CI: 0.75, 2.06; Model 3: β = 0.77, CI: 0.03, 1.51). In contrast, among males, Black race was not associated with BMI in any model. Hispanic ethnicity was not associated with BMI among females in either Model 2 or 3, while among males the effect sizes were similar in Models 2 and 3.

#### The change in between school variance with the addition of individual and school characteristics (addressing Objective 3)

Among females and males, the unconditional model (Model 1) indicated significant variation in BMI between schools, as reflected in the sigma_u values for both models. These values are the square roots of school-level variance components and can be interpreted as school-level standard deviations, so that the amount by which a typical school differs from the overall average BMI is about 1 unit (1.34 units for females, 0.97 units for males). In contrast, the standard deviation at the student level is approximately 5 units for both males and females. In females, after controlling for race/ethnicity, age, household demographics, and parental BMI, the between-school variation in BMI was no longer statistically significant and was estimated at 0 after accounting for chance variation at the student level. In males, the inclusion of individual level characteristics did not fully explain the between school variation in BMI among males, although their inclusion did result in an approximate one-third reduction in between-school variance (sigma_u^2^) (see Table 3, Model 2). The addition of school characteristics and programs did not further decrease the between-school variance in BMI.

#### Additional findings

Black females had BMIs averaging 1.4 kg/m^2^ higher than White females. Mean BMI did not significantly differ from Whites for any other female racial/ethnic group. Females with an obese/overweight parent or caregiver had BMIs averaging >2 kg/m^2^ higher than those who did not have an obese/overweight parent or caregiver.

Hispanic males averaged nearly 1.5 kg/m^2^ higher in BMI than their White peers. Both markers of socioeconomic status—household income and highest educational level achieved in the household—had inverse but non-significant relationships with BMI. Males with an obese or overweight parent or caregiver had BMIs averaging >2 kg/m^2^ higher than those who did not have an obese/overweight parent or caregiver.

## Discussion

In our study of a multi-ethnic cohort of 5^th^ graders in Los Angeles, Birmingham, and Houston, we found that females attending higher income schools had lower average BMIs than females attending lower income schools. While there were significant disparities in BMI between White and Black females, this difference was largely explained by school characteristics; the difference in Black and White female BMI was reduced by half with adjustment for school factors. With respect to school-level differences in BMI, individual characteristics (i.e., compositional factors), not school factors (i.e., contextual factors), explained all of the between-school variance in female student weight status and a large portion in males. This study found no associations between school physical activity or nutrition resources/programs and individual BMI in this racially diverse cohort of 5^th^ graders. None of the 10 school-level nutrition and physical activity resources or the summary score was associated with BMI in either males or females.

Our findings are in concert with those of Schuster et al. [8] in which disparities in a number of health behaviors and outcomes were noted to be attenuated by school characteristics. Our findings, however, demonstrate that for obesity it is specific to the disparity in weight status between Black and White females.

Unlike prior studies [6], [7], we were able to explain all of the between-school variation in weight status among females by accounting for the student makeup. Much of the variance in males was similarly explained by compositional factors, not contextual factors. In a prior analysis of the National Longitudinal Study of Adolescent Health (Add Health), a nationally representative school-based study of adolescents, Richmond and Subramanian found a 50% reduction in the between-school variability in student BMI when controlling for student- and school-level demographics; however, significant variation in BMI between schools remained for both males and females.(6) Similarly, O'Malley et al. found persistent between-school variation in weight status in the Monitoring the Future high school cohort.(7) Both Add Health and Monitoring the Future have older cohorts (mostly high school aged) and neither has data regarding the weight status of the parents, which may explain the difference in the results.

Having a parent/caregiver who is overweight/obese was associated with notably higher BMI in both males and females, similar to findings from other studies [11], [12]. Parental/caregiver obesity likely influences the students' weight status through multiple mechanisms including, but not limited to, genetic factors, nutritional patterns and role modeling, and physical activity behaviors. The weight status of the caregiver may also reflect the influences of the neighborhood or home environments, influences not accounted for in this analysis.

The lack of statistical association between school nutrition and physical activity resources/programs is not surprising in this cohort and is consistent with findings from other studies. Because the school-level variability was largely explained by individual factors, there was little between-school variability remaining to be explained by school-level resources or programs. These findings echo those from other studies. Terry-McElrath and colleagues found minimal associations between the school food environment and student BMI [13]; in another study using the same cohort they found little association between participation in PE and/or sports and student weight status [14]. In contrast, Fox et al. found promising results when they looked at serving specific menu items and student BMI [15].

### Limitations

There are limitations to this study that should be noted. First, the findings can only be generalized to similar urban populations attending similar schools. Second, our summary score does not capture how physical activity and nutrition programs were actually implemented and the degree of student participation. It is possible that schools that scored similarly on the physical activity/nutritional resources summary score could differ considerably in the quality and participation levels in their programs. Finally, given the cross-sectional nature of the study, no causal inference can be made regarding the findings.

### Conclusions

We find that school characteristics and programs largely explain Black/White disparities in female weight status and that school SES is inversely related to female weight status. However, school nutrition and physical activity programming were not associated with individual weight status in males or females. This study is unable to compare the effects of any school programs vs. none but instead demonstrates that there is no differential impact on BMI when programs in one school are compared with another, even in schools of different incomes and racial/ethnic makeup. The study highlights the need to understand how school programs such as the school lunch program and/or PE are actually implemented and utilized by students. This may allow future researchers to shed light on the influence of different types or intensities of programs and thus identify programs with the greatest promise of impacting student BMI. In the meantime, schools should be viewed as a potential venue in which to intervene on the commonly seen disparities in female BMI.

### Humans Subject Approval Statement

All analyses were approved by the Boston Children's Hospital Institutional Review Board.
